# Outcomes for Efavirenz versus Nevirapine-Containing Regimens for Treatment of HIV-1 Infection: A Systematic Review and Meta-Analysis

**DOI:** 10.1371/journal.pone.0068995

**Published:** 2013-07-22

**Authors:** Prinitha Pillay, Nathan Ford, Zara Shubber, Rashida A. Ferrand

**Affiliations:** 1 Wits Reproductive Health and HIV Institute, Faculty of Health Sciences, University of the Witwatersrand, Johannesburg, South Africa; 2 Médecins Sans Frontières, Johannesburg, South Africa; 3 London School of Hygiene and Tropical Medicine, London, United Kingdom; 4 Centre for Infectious Disease Epidemiology and Research, University of Cape Town, Cape Town, South Africa; 5 Department of Infectious Disease Epidemiology, Faculty of Medicine, Imperial College, London, United Kingdom; Boston University, United States of America

## Abstract

**Introduction:**

There is conflicting evidence and practice regarding the use of the non-nucleoside reverse transcriptase inhibitors (NNRTI) efavirenz (EFV) and nevirapine (NVP) in first-line antiretroviral therapy (ART).

**Methods:**

We systematically reviewed virological outcomes in HIV-1 infected, treatment-naive patients on regimens containing EFV versus NVP from randomised trials and observational cohort studies. Data sources include PubMed, Embase, the Cochrane Central Register of Controlled Trials and conference proceedings of the International AIDS Society, Conference on Retroviruses and Opportunistic Infections, between 1996 to May 2013. Relative risks (RR) and 95% confidence intervals were synthesized using random-effects meta-analysis. Heterogeneity was assessed using the I^2^ statistic, and subgroup analyses performed to assess the potential influence of study design, duration of follow up, location, and tuberculosis treatment. Sensitivity analyses explored the potential influence of different dosages of NVP and different viral load thresholds.

**Results:**

Of 5011 citations retrieved, 38 reports of studies comprising 114 391 patients were included for review. EFV was significantly less likely than NVP to lead to virologic failure in both trials (RR 0.85 [0.73–0.99] I^2^ = 0%) and observational studies (RR 0.65 [0.59–0.71] I^2^ = 54%). EFV was more likely to achieve virologic success than NVP, though marginally significant, in both randomised controlled trials (RR 1.04 [1.00–1.08] I^2^ = 0%) and observational studies (RR 1.06 [1.00–1.12] I^2^ = 68%).

**Conclusion:**

EFV-based first line ART is significantly less likely to lead to virologic failure compared to NVP-based ART. This finding supports the use of EFV as the preferred NNRTI in first-line treatment regimen for HIV treatment, particularly in resource limited settings.

## Introduction

According to the 2010 World Health Organisation (WHO) HIV treatment guidelines [1], the choice of non-nucleoside reverse transcriptase inhibitor (NNRTI) for first-line antiretroviral therapy (ART) for HIV-1 infected adults is either efavirenz (EFV) or nevirapine (NVP), in combination with either zidovudine (AZT) or tenofovir (TDF) and lamivudine (3TC) or emtricitabine (FTC). In contrast, the US Department of Health and Human Services [2] and the International AIDS Society US guidelines [3] recommend a preference for EFV over NVP for first-line therapy. More recently, WHO has recommended that EFV should be considered as the preferred first-line NNRTI [4]. A previous Cochrane review concluded that there was no difference in efficacy between the two drugs but found a higher risk of acquired resistance for patients on NVP [5]. This finding was dominated by the large 2NN Study comparing NVP and EFV regimens that found no difference in efficacy between the two drugs [6]. A more recent review comparing the use of these drugs specifically with TDF-containing regimens concluded that EFV had superior virological efficacy [7].

In order to provide evidence in support of future regimen choice, this systematic review provides an updated assessment of the evidence regarding comparative efficacy of these two NNRTI drugs as part of first-line antiretroviral therapy.

## Methods

### Criteria for Considering Studies for this Review

#### Types of studies

This review considers both experimental and epidemiological study designs, including randomized controlled trials (RCTs), non-randomised controlled trials, quasi-experimental, before and after studies, prospective, retrospective and comparative cohort studies, and analytical cross-sectional studies for inclusion.

#### Types of participants

This review considered studies that included HIV-1 infected individuals who have not been previously exposed to combination ART. For studies that include participants irrespective of previous exposure, only data from ART-naive patients were extracted. Exclusions included pregnant women, ART experienced patients, virological failure (rebound) in patients previously suppressed, where no viral load measurements were done, and studies with planned switching to EFV or NVP.

#### Type of interventions

This review included studies that evaluated EFV as compared to NVP-containing regimens in a combination of three antiretroviral drugs only. The triple drug combination therapy must contain two NRTIs with either EFV or NVP. If cohorts report on other drugs in combination with EFV or NVP, or two NRTIs and a protease inhibitor, then only data for combination ART of two NRTIs with NVP or EFV were extracted.

#### Types of outcome measures

This review considers studies that included the following outcome measures:

### 1. Primary Outcomes

Virologic outcomes: comparison using plasma HIV-1 RNA levels as measure of efficacy. Success was defined as HIV-1 RNA plasma levels less than a value (copies/ml) as defined by the authors/studies. Failure was defined as HIV-1 RNA plasma levels more than a value (copies/ml) specified by the authors/studies. If several time points are reported, data from the last point of analysis was used.

### 2. Secondary Outcomes

Treatment termination/discontinuation (any cause) and mortality were sought.

### Search Strategy

(See Table S1 for details of search strategy).

A preliminary search of PubMed and Embase was undertaken to identify key text words contained in the titles and abstracts of relevant articles, and of the index terms used to describe an article. A second search, using all identified keywords and index terms, was then undertaken across the following databases: PubMed, Embase and Cochrane Central Register of Controlled Trials (Central). The bibliographies of all 139 full text reports and articles were searched for additional studies. No language or geographical restriction was applied. Finally, the abstract database of all conferences of the International AIDS Society and the Conference on Retroviruses and Opportunistic Infections was searched. Studies published from January 1996 (the advent of triple combination ART) to 01 May 2013 were considered for inclusion in this review. All titles and abstracts were reviewed, duplicates excluded and articles meeting the pre-defined inclusion criteria were selected.

### Data Extraction and Analysis

Data were extracted into pre-piloted Microsoft Excel tables and included details about the interventions, populations, study methods and outcomes of significance. Key outcome data extractions were verified by duplicate extraction. Data analysis was conducted using RevMan version 5.0 [8]. Papers selected for review were assessed for risk of bias according to the following criteria: random sequence generation (selection bias), allocation concealment (selection bias), blinding of participants and personnel (performance bias), blinding of outcome assessment (detection bias), selective reporting (reporting bias),comparability of baseline groups, application of intent-to-treat analysis, and proportion lost-to follow up (see Table S2). Quality assessment on design of study, risk of bias, inconsistency, indirectness and imprecision were assessed using the GRADE framework [9] (www.gradeworkinggroup.org). Where sufficient studies were available, publication bias was assessed visually using funnel plots.

Relative risks (RR) for primary and secondary outcomes were calculated on an intent-to-treat basis and pooled using random effects meta-analysis. Where statistical pooling was not possible or deemed inappropriate, study-specific outcomes are presented.

Heterogeneity was examined using the χ^2^ statistic with a significance level of >0.10, and the I^2^ statistic with an I^2^ estimate greater than 50% was considered indicative of moderate to high levels of heterogeneity [10]. The DerSimonian-Laird random-effects method was used to recognize and anchor studies as a sample of all potential studies, and to incorporate an additional between-study component to the estimate of variability. If significant statistical heterogeneity was found, and where feasible, subgroup analyses were done to explore differences in outcomes according to study design, duration of follow up, virological failure or success as reported by the studies, studies for patients on tuberculosis (TB) treatment, and study setting. Sensitivity analysis explored the potential influence of NVP dosing schedule (200 mg twice daily and 400 mg once daily) and differing thresholds of virologic failure.

## Results

### Description of Studies

**Figure 1:**
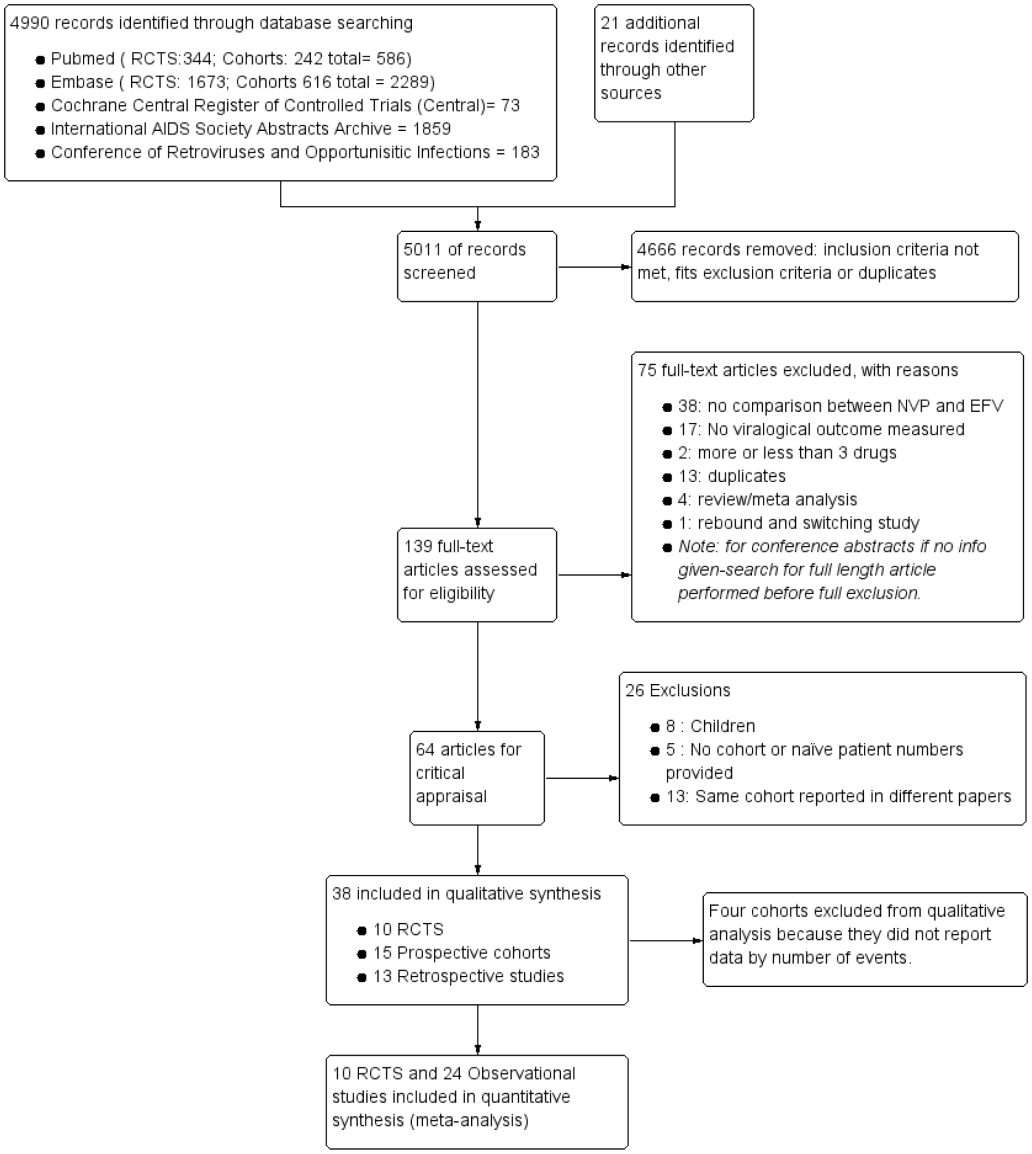
Search strategy.

The search yielded 4990 abstracts, with 21 additional articles identified from references of key articles. One hundred and thirty nine articles were reviewed in full and 38 were included in the final analysis (Fig. 1). In total, this review includes virologic outcome data from 114,391 HIV-I-infected, combination ART-naive patients from 27 countries. Most of the virological outcome data are from high-income resource-rich settings and only eleven published papers reported data from resource-limited settings (South Africa, Nigeria, Senegal, Zambia, Botswana, Zimbabwe, Uganda, Thailand, Mozambique, Burkina Faso and India). The final included studies comprised of 10 randomised trials (data from 11 articles with additional long term data on the 2NN study [6] from a second publication [11]); 15 prospective cohorts and 13 retrospective cohorts. One RCT [12] also reported on a non-randomised cohort, but this cohort was not included as the outcomes were not disaggregated by the NRTI backbone.

RCTs contributed 2% of total patient data, prospective cohort studies contributed 57% of data, and the remainder (41%) came from retrospective cohorts. In total, 63% of patients were on EFV.

The majority of studies compared EFV 600 mg once daily against NVP 200 mg twice daily. One study adjusted EFV dose to weight [13], and two studies used NVP 400 mg once daily [14], [15]. Fifteen studies did not report NVP dosage, and were all assumed to use 200 mg twice daily as this is the standard recommended dosage [1].

NRTI backbones used differed between studies. Stavudine (d4T)/3TC were used in 21 studies and 9 studies did not use this NRTI backbone at all. AZT/3TC was used in 21 studies and 9 studies did not use this backbone at all. TDF/3TC or TDF/FTC was used less frequently, in only 7 studies. Seven studies did not report on NRTI backbones used.

Sample sizes ranged from 50 patients [16] to >27,000 patients [17]. Over half of all studies (n = 20) were published after 2008.

Overall, more females were likely to be on NVP as EFV use has, until recently, been contra-indicated in pregnancy [18]. Only one study, the HIV-CAUSAL collaboration [19], excluded those with AIDS-defining illness. Baseline characteristics are summarized in Table 1.

**Table 1:** Baseline characteristics of patients on NVP and EFV by study design.

Randomised controlled trials															
Study and Cohort	Setting and Total patients in Cohort	Nevirapine (NVP)						Efavirenz (EFV)						Time point of analysis (weeks)	HIV Virologic outcome definition
		Median Age	Female %	Median Baseline CD4 cells/mm3	Median Baseline viral load copies/ml Or log10c/ml	% with AIDS or CDC/C	Total on NVP	Median Age	Female %	Median Baseline CD4 cells/mm3	Median Baseline viral load copies/ml or log10c/ml	% with AIDS or CDC/C	Total on EFV		
**Wit ** ***et al*** ** (2007) 2NN** [**11**]	North & South America, Australia, South Africa, Europe, Thailand (n = 567)	34	39%	170	4,7	22%	224	33	32%	190	4,7	18,7%	223	49–144	VF>50
**van den Berg-Wolf ** ***et al*** ** (2008) CPCRA** [**12**]	US (n = 228)	36	22%	196	5.1	37%	117	38	23%	181	5.0	38%	111	>32	VF>1000 VS<50
**Nunez ** ***et al*** ** (2002) SENC** [**14**] 1	Madrid, Spain (n = 67)	35	22%	353	23952	6%	36	35	23%	416	22789	16%	31	@48	VF>50 VS<50
**Swaminathan ** ***et al*** ** (2011)** [**15**] **^1^**	Southern India (n = 116)	38	23%	83	282000	–	57	34	17%	85	362000	–	59	@24	VF>400
**Manosuthi ** ***et al*** ** (2009) N2R** [**20**] 2	Thailand (n = 142)	38	31%	56	5.75		71	36	33%	75	5.75		71	@48	VS<50 VF>1000
**Bonnet ** ***et al*** ** (2013) CARINEMO** [**21**] **^2^**	Mozambique (n = 570)	–	–	92	5.5	–	285	–	–	86	5.6	–	285	@48	VS<50
**Matteelli et al (2013)** [**22**] **^2^**	Burkina Faso (n = 69)	38	51%	–	–	–	33	38	515	–	–		36	@24 &@48w	–
**Wester ** ***et al*** ** (2010) TSHEPO** [**27**]	Gaborone, Botswana(n = 658)	33	71%	199	183000	7,7%	325	33	68%	199	204000	10,2%	325	>16	>5000 &>400^3^ VS<50
**Ayala Gaytan ** ***et al*** ** (2004)** [**38**]	Mexico (n = 58)	32	18%	143	–	71%	24	36	20%	131	–	83%	19	@24 & @48	VS<400
**Landman ** ***et al*** ** (2011) DAYANA** [**39**]	Cameroon, Senegal (n = 110)	39	*66%*	*200*	*5,4*	*–*	31	*39*	*66%*	*200*	*5,4*	–	30	@16 & @48	VS<50

Prospective Cohorts															
Study and Cohort	Setting and Total patients in Cohort	Nevirapine (NVP)						Efavirenz (EFV)						Time point of analysis (weeks)	Virologic outcome definition
		Median Age	Female %	Median Baseline CD4 cells/mm3	Median Baseline viral load copies/ml or log10c/ml	% with AIDS or CDC/C	Total on NVP	Median Age	Female %	Median Baseline CD4 cells/mm3	Median Baseline viral load copies/ml or log10c/ml	% with AIDS or CDC/C	Total on EFV		
**Perez-Elias ** ***et al*** ** (2005)** [**16**]	Spain (n = 100)	*38*	*27.5%*	*312*	*4.9*	*17,6%*	23	*38*	*27,5%*	*312*	*4,9*	*17,6%*	28	@48	VS<50
**Fox ** ***et al*** ** (2012) IDEA** [**17**]	South Africa (n = 19645)	*–*	*66%*	*93*	*65000*	*20%*	5243	*–*	*66%*	*93*	*65000*	*20%*	14402	>24	VF>1000
**HIV-CAUSAL (2012) ** [**19**]	USA, UK Netherlands France, Switzerland, Spain, Greece, France,(n = 19863)	–	31.9%	–	–	–	8129	–	21,7%	–	–	–	15336	@48	VF>400
**Keiser ** ***et al*** ** (2002)** [**40**]	US (n = 1078)	37	18%	310	3.87	–	523	37	17%	264	4.37	–	555	48–96	VS<400
**Annan ** ***et al*** ** (2009)** [**41**]	Chelsea, UK (n = 994)	37	20%	*173*	*4.9*	–	271	38	12%	*173*	*4.9*	–	723	>12–24	VS<500
**Reekie ** ***et al*** ** (2011) EUROSIDA** [**42**]	Europe, Israel, Argentina (n = 603)	40	29,8%	359	2.7	24,2%	166	40	24,8%	323	3.59	25,2%	561	24–192	VS<500
**Hamers ** ***et al*** ** (2012) PASER** [**43**]	Kenya, Nigeria, South Africa, Uganda, Zambia, Zimbabwe (n = 2579)	*38*	*58%*	*133*	*5*	*17%*	1037	*38*	*58%*	*133*	*5*	*17%*	1542	@48	VF>400
**Cortes ** ***et a*** **l (2010)** [**44**]	Chile (n = 5120)	NR	-	151	–		1080	–		86	–	–	3107	@192	VS<80
**Castelnuovo ** ***et*** **** ***al*** ** (2011)** [**45**]	Kampala, Uganda (n = 559)	*34*	*69%*	*107*			350	*34*	*69%*	*107*			123	24–192	VF>1000
**ART CC (2006) ** [**46**]	20 countries in Europe, Argentina, USA, UK, Canada,(n = 17666)	36	–	293	4.7	12%	2378	36	–	225	4.9	22%	2254	@ 24	VF>500
**Mugavero (2008) ART CC(b) ** [**47**]	20 countries in Europe, Argentina, USA, UK, Canada (n = 13546)	36	39%	260	4.7	12%	2151	39	22%	207	4.9	22%	3788	@24	VF>500
**Hartmann ** ***et al*** ** (2005) NEEF** [**48**]	Germany (n = 622)	NR	23%	317	4.18	19%	86		27%	263	4,73	15%	52	@48	VS<50
**Manfredi ** ***et al*** ** (2004)** [**49**]	Italy (n-662)	35	38%	365	4.4	12%	73	35	36%	345	4,5	14%	81	24–48	VS<50
**de Beaudrap ** ***et al*** ** (2008) ANRS 1215/1290** [**50**]	Senegal (n = 404)	36	28%	189	4.94	38%	47	37	48%	158	5,28	56%	170	**–**	VS<500
**Wallis ** ***et al*** ** (2012) CIPRA-SA** [**51**]	South Africa (n = 812)	–	*71%*	*164*	*5.1*	*34,7%*	153	*–*	*71%*	*164*	*5,1*	*34,7%*	597	>24	VF>1000

Retrospective Cohorts															
Study and Cohort	Setting and Total patients in Cohort	Nevirapine (NVP)						Efavirenz (EFV)						Time point of analysis (weeks)	Virologic outcome definition
		Median Age	Female %	Median Baseline CD4 cells/mm3	Median Baseline viral load copies/ml or log10c/ml	% with AIDS or CDC/C	Total on NVP	Median Age	Female %	Median Baseline CD4 cells/mm3	Median Baseline viral load copies/ml or log10c/ml	AIDS or CDC/C%	Total on EFV		
**Villar ** ***et a*** **l (2011)** [**13**] ^2^	Barcelone, Spain (n = 63)	–	*16%*	*86*	*5,3*	*–*	13	*–*	*16%*	*86*	*–*	*–*	50	@48	VF>50
**Shipton ** ***et al*** ** (2009) Masa** [**23**] ^2^	Gaborone, Botswana (n = 155)	32	96%	81	506486	–	130	39	35%	76	521843	–	180	12–48	VS<400
**Manosuthi ** ***et al*** ** (2008)** [**24**] ^2^	Thailand (n = 188)	35	26%	36	5,7	–	111	36	30%	36	5,6	–	77	@24	VS<50
**Amoroso ** ***et al*** ** (2012) AIDSRelief** [**25**]	Kenya, Uganda, Zambia, Nigeria (n = 3862)	*38*	–	–	–	–	2209	*38*	–	–	–	–	1703	–	VS<400
**Bock ** ***et al*** ** (2010)** [**26**]	South Africa (n = 19441)	36	59,8%	107	–	–	7909	31	88,6%	132	–	–	19441	@24	VS<400
**Cozzi-Lepri ** ***et al*** ** (2002) ICONA** [**28**]	Italy (n = 694)	35	35%	423	4,6	2,9%	171	35	28,7%	307	4,7	20,2%	94	24–72	VF>500
**Braithwaite ** ***et al*** ** (2007) ‘Veterans’** [**33**]	Veterans Affairs Healthcare System, US (n = 6394)	46	1,8%	313	4,5	–	512	47	2,8%	232	4,8	–	1140	24–72	VS<500
**Nachega ** ***et al*** ** (2008)Aid for Aids** [**34**]	South Africa (n = 2817)	36	67,5%	171	5,1	–	995	37	60,3%	136	5,2	–	1822	24–96	VF>400
**Chung and Fichtenbaum (2002) ** [**49**]	USA (n = 1664)	*35*	*13%*	*155*	*81655*	–	NR	*35*	*13%*	*155*	*81655*	–	–	@24	VS<400
**Darin ** ***et al*** ** (2010) PEPFAR Nigeria** [**52**]	Nigeria (n = 8418)	35	*62%*	*142*	*–*	*–*	6254	*35*	*62%*	*142*	*–*	–	2164	@48	VF>1000
**von Giesen ** ***et al*** ** (2003)** [**53**]	Germany (n = 733)	42	22%	–	–	92%	131	42	19,6%	–	0	68%	132	–	VF>10000
**Cescon ** ***et al*** ** (2010) CANOC** [**54**]	British Columbia, Ontaria and Quebec, Canada (n = 3555)	40	*80%*	*–*	*–*	*13%*	675	*40*	*80%*	*–*	*0*	*13%*	952	>48	VS<50
**Manosuthi ** ***et al*** ** (2004)** [**55**] ^2^	Bangkok (n = 66)	–	41,7%	–	–	–	30	–	27,6%	–	–	–	36	–	VS<400

1 These two studies compared NVP 400 mg once daily to EFV 600 mg once daily. All other studies compared NVP200 mg twice daily to EFV 600 mg once daily.

2 Studies in TB/HIV co-infected patients on TB treatment.

3 after July 2007, the definition of failure changed from more than 5000copies/ml to more than 400copies/ml.

Abbreviations: EFV efavirenz NVP nevirapine Dash (–) Not provided VS Virologic suppression VF Virologic failure Italics: Overall cohort characteristics not differentiated by NNRTI.

Six studies were done exclusively in TB/HIV co-infected patients [13], [20], [21], [22], [23], [24]. Another study included 188 patients on EFV and 86 patients on NVP who were co-infected with TB [25] while 36.1% of the IeDEA cohort [17] and 6.7% of the Kheth’Impilo cohort [26] were TB co-infected. These studies do not report the virologic outcomes of those co-infected patients and were thus not included in that subgroup meta-analysis.

### Risk of Bias and GRADE Assessment

The assessment of the overall quality of the studies is summarised in . Only two trials reported on allocation concealment [6], [15], and all studies were open label. Three of the randomised studies were partly or fully funded by the pharmaceutical industry [11], [12], [27], and this was disclosed in their publications; the others did not report on their source of funding (see Table S2). Two observational studies took a random selection from the observational cohort for their analysis [23], [25]. Only 13 studies (five of ten RCTs and eight observational cohorts) reported loss to follow-up figures and all were below 20% (0.5% to 19.8%) (Table S2).

The evidence from RCTs was considered to be high quality for critical outcomes: there were no evidence of serious risk of bias, inconsistency, imprecision or indirectness In contrast, the evidences from observational studies was judged to be of very low quality, mainly due to risk of bias (lack of random sampling, baseline imbalances, and retrospective design), and inconsistency in the direction and imprecision in the confidence intervals around the point estimates. There were some well-designed prospective cohort and collaborative cohort studies that were rated to be of moderate quality, but this was not sufficient to upgrade the quality of the observational data overall.

**Figure 2:**
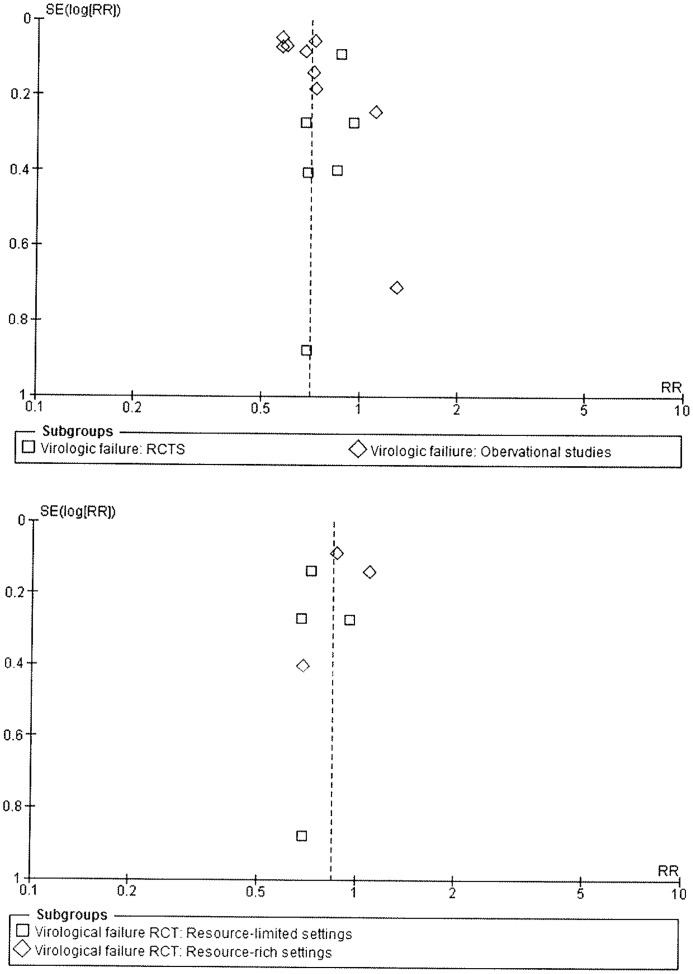
Funnel Plots.

Publication bias was assessed by funnel plot (Fig. 2) and the eggers test for small study effects; these analyses were limited to the primary outcomes of virologic failure and success for observational studies because there were too few RCTs to allow these analyses to be performed. No significant bias was detected for either outcome (p = 0.2).

### Virologic Failure

**Figure 3:**
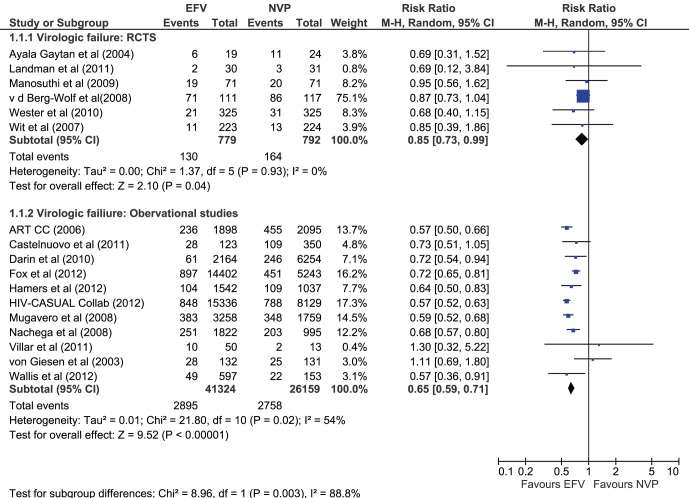
Outcome virologic failure in RCTs and observational studies.

Six RCTs (n = 1572) provided evidence for the primary outcome of virologic failure. Overall, 16.7% on EFV and 20.7% of NVP patients failed treatment (RR 0.85 [0.73– 0.99], I^2^ = 0%) (Fig. 3). This result was consistent for the estimates derived by pooling data from nine observational studies (n = 67483): 7% of patients taking EFV versus 10.5% of those on NVP were observed to have failed treatment (RR 0.65 [0.59–0.71]) (Fig. 3). There was significant heterogeneity between studies (I^2^ = 54%), which was largely explained by the inclusion of the large, combined cohorts of IeDEA [17] and the HIV CAUSAL Collaboration [19].

### Virologic Success

**Figure 4:**
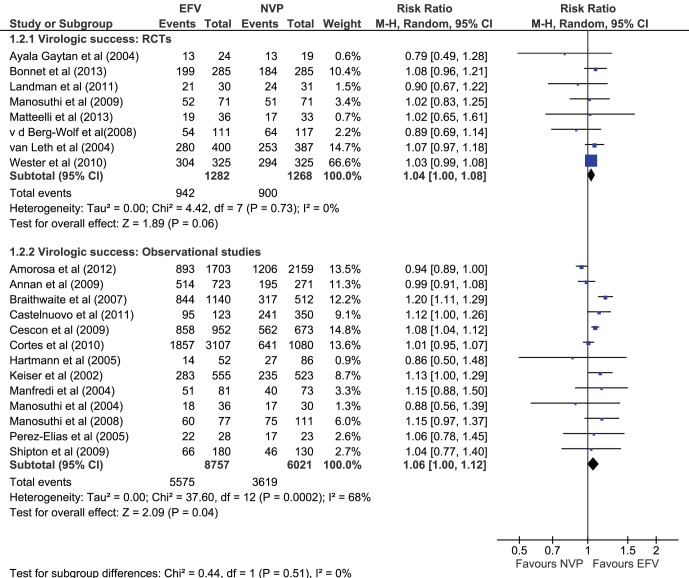
Outcome virologic success in RCTs and observational studies.

There was a marginal significance between the two drugs, with EFV being more likely to achieve virological success compared to NVP. Eight RCT (n = 2550) that measured virologic success (HIV-1 RNA copies/ml less than a specified cut-off value) showed that patients on EFV (73.7%) were more likely to achieve success than those taking NVP (200 mg twice daily) (70.4%) with a pooled RR of 1.04 [95%CI 1.00–1.08], and no heterogeneity between the studies was observed (I^2^ = 0%) (Fig. 4). Observational studies that reported on success (13 of 28; n = 14778) also reported better rates of suppression; 63.7% for EFV versus 60.1% for NVP with a pooled RR of 1.06 [1.00– 1.12] (Fig. 4). Heterogeneity was moderate (I^2^ = 68%) and in subgroup analysis this appeared to be largely explained by the inclusion of retrospective studies.

Four cohorts did not report data by number of events and thus were not included in the meta-analysis but their findings are consistent with the overall result. One study reported no difference in virologic success between the two drugs (HR 1.37 [0.35–1.68] [36]. Bock and colleagues reported that those on EFV were more likely to suppress (<400copies/ml) (adjusted odds ratio 1.29 [1.05–1.59]) [26]. Another study found that patients on NVP were more likely to fail (adjusted hazards ratio 2.15 [0.90–5.13]) [28]. Finally, a fourth study reported that virologic success at six months was 38% for NVP and 59% for EFV-based ART, although loss to follow up in this study was highly differential (25% for NVP and 41% for EFV) [29].

### Virologic Outcomes in HIV/TB Co-infected Patients

Six studies provided data on virologic success of TB/HIV co-infected patients on TB treatment (n = 1187). Those on EFV and TB treatment were no more likely to suppress than those on NVP in three RCTs (RR = 1.06 [0.97– 1.17] = 0%) and in three comparative cohort studies (RR 1.02 [0.70–1.47] I^2^-63%). Four observational studies provided data on virologic failure with those on EFV and TB treatment more likely to suppress than those on NVP (RR 0.58 [0.34, 0.99] I^2^ = 78%).

### Mortality

**Figure 5:**
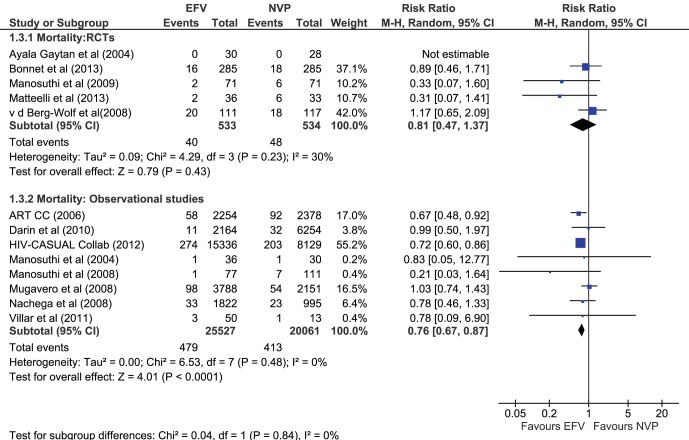
Outcome mortality in RCTs and observational studies.

There was no significant difference noted in mortality rates in four RCTs between the two NNRTI’s (n = 1067) (RR of 0.81 [0.47, 1.37] I^2^ = 30%). However, in the eight observational studies that reported mortality (n = 45588), EFV was protective (RR 0.76 [0.67–0.87], I^2^ = 0%) compared to NVP (Fig. 5).

### Treatment Discontinuation (any cause)

Date from five RCTs (n = 1648) showed no significant difference between EFV and NVP in terms of discontinuation of treatment due to any cause (RR 0.83 [0.55–1.25]). Similar results were found in seven observational studies (RR = 0.89 [0.73–1.08]) with 27% in both NNRTI groups alike discontinuing treatment for any reason. The majority of treatment discontinuations were driven by adverse events.

### Sensitivity Analysis

A sensitivity analysis was carried out to assess if the results of the meta-analysis are robust depending on the different dosages of NVP and differing threshold definitions of failure. The results show no significant difference of the relative risk of an outcome when EFV was compared strictly to studies of NVP 200 mg twice daily [14], [15], compared to studies regardless of NVP dosage (Table S3).

Sensitivity analyses results of the meta-analysis, after excluding studies that used a lower threshold and observing how this affected the results, showed that the risk of failure for those taking NVP is consistently much higher than EFV irrespective of different thresholds in both RCTs and observational studies (Table S3).

### Subgroup Analysis

**Figure 6:**
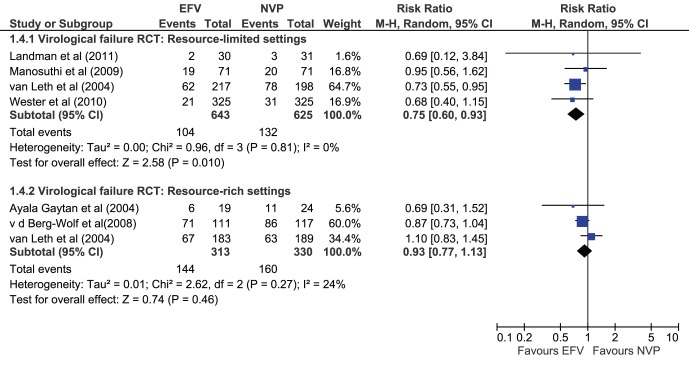
Outcome virologic failure in RCTs-subgroups by settings.

Several subgroup analyses were performed to assess the potential influence of study duration. For the outcome of virological suppression, studies that ran to 24 weeks were found to be non-significant (RR 1.15, 95% CI 0.89–1.50) although this is likely due to small sample size as only 2 studies contributed to this subgroup analysis; for 48 weeks, the results remained significant (8 studies: RR 1.04, 95%CI 1.00–1.08). In subgroup analyses of different settings, patients on EFV compared to NVP in RCTs were less likely to fail in resource-limited settings (RR 0.75 [0.60, 0.93] I^2^ = 0%) but not in resource-rich settings (RR 0.93 [0.77–1.13] I^2^ = 24%) (Fig. 6).

## Discussion

This systematic review found EFV was significantly less likely to lead to virological failure than NVP. Although marginally significant, EFV was also more likely to achieve virological success than NVP. These findings were consistent across all study designs. Among TB/HIV co-infected patients, there was no difference in viral suppression among those on EFV and TB treatment and those taking NVP and TB treatment. Mortality appeared to be lower among patients on EFV in observational studies, but this was not seen in the RCTs.

Based on the large 2NN RCT [6], which found similar efficacy between EFV and NVP to suppress HIV-1 levels below 50 copies/ml but significant differences in virologic failure by region, a subgroup analysis was performed on RCTs comparing those conducted within resource-limited settings to those conducted in resource-rich settings. This analysis found that the benefit of EFV over NVP was especially highlighted in resource-limited settings when compared to resource-rich settings. This is important for resource-limited settings where the smaller risk of EFV leading to treatment failure has a critical effect in reducing the risk of unnecessary switches to a more expensive second-line treatment.

Viral load measurements are not widely available in resource-limited settings. The sensitivity analyses showed that regardless of the threshold definition of virologic failure used, EFV consistently proves to be a better option. If treatment aims for viral suppression are to avoid the emergence of resistance, disease progression and death, then patients should be initiated on a more robust, durable first-line NNRTI such as EFV, especially in resource-limited setting where alternative options are limited.

A previous systematic review compared 7 RCTs (1,688 patients) of EFV and NVP use in treatment-naïve individuals and found no critical difference between the regimens [5]. This review includes additional data from 3 further RCTs, and data from observational studies which was able to assess outcomes among a total of 114 391 patients. Furthermore, we included updated, longer term outcome reports from the largest RCT (the 2NN study [6], [11]). This review also limited all analyses to ART-naïve patients and to those who had two NRTIs as a backbone, in contrast to the previous review which also included patients receiving protease-inhibitor based therapy. Another recent review that assessed comparative efficacy of EFV compared to other regimens also found superior virologic suppression in favour of EFV-based regimen [30]. Our review differed from this review by focusing specifically on the NVP versus EFV studies, thereby including a much larger dataset for this comparison.

There are several limitations to this review. First, we chose to include observational data in order to assess a wider evidence base, but observational studies are subject to unmeasured confounding. To address these concerns we presented trial and observational data separately, and undertook subgroup analyses to explore the potential influence of study design on our primary outcome, and no important differences were found. Second, differences in virological outcomes may be partly explained by differences in adherence between the groups because in some studies the EFV-containing regimen was administered as a once-a-day regimen, and EFV is associated with a lower overall frequency of adverse events [31]; both of these issues are associated with improved adherence. However, while once-daily dosing improves adherence the overall effect on virologic suppression is unclear [32], and in this review studies that have adjusted for adherence still found a better virological response with EFV [33], [34], [35]. As with any systematic review, another limitation is publication bias. Attempts were made to limit the possibility of having missed studies by including conference abstracts, and trying to contact authors for more information, and there was no statistical evidence of publication bias. Differential LTFU between intervention groups is an important source of bias. This was poorly reported by studies, but in 6 studies where LTFU was reported by drug, this appeared to be non-differential. Lastly, we could not explore the potential influence of differing NRTI backbones as too few studies provided data of outcomes by backbone that were not already accounted for in two previously published reviews [5], [7]. The Cochrane review reported that both EFV and NVP have demonstrated clinical efficacy largely with patients on a d4T/3TC NRTI backbone [5], the majority of whom were drawn from the 2NN study [6]. However, a more recent meta-analysis showed that even with newer regimens containing TDF, NVP was inferior to EFV [7].

Future studies are encouraged to report data for both treatment success and failure, using internationally agreed definitions, and important secondary outcomes.

In conclusion, the findings of this review as well as recent recommendations to use EFV in the first trimester of pregnancy [4], [36], its improved toxicity profile [31], and improved cost-effectiveness resulting from recent EFV price reductions [37]; all support recommendations preferring the use of a once daily fixed-dose combination of TDF/3TC/EFV.

## Supporting Information

Table S1 **Search strategy details.**
(DOC)

Table S2 **Risk of bias assessment.**
(DOCX)

Table S3 **Sensitivity analysis results.**
(DOCX)

Table S4 **PRISMA Checklist.**
(DOC)
