# Supplementary material for: Outcomes for Efavirenz versus Nevirapine-Containing Regimens for Treatment of HIV-1 Infection: A Systematic Review and Meta-Analysis
Source: PLoS One. 2013 Jul 22;8(7):e68995. doi: 10.1371/journal.pone.0068995 (PMC3718822; doi:10.1371/journal.pone.0068995)
Supplement: Table S1 — Search strategy details. (DOC) [file pone.0068995.s001.doc]

**Table S1: Search strategy details**

| **PubMed search string results** | | |
| --- | --- | --- |
| **#1** | Search ((SearchHIVInfections[MeSH] AND ORHIV[MeSH] AND ORhiv[tw] OR hiv-1*[tw] OR hiv-2*[tw] OR hiv1[tw] OR hiv2[tw] OR hiv infect*[tw] OR human immunodeficiency virus[tw] OR human immunedeficiency virus[tw] OR human immuno- deficiency virus[tw] OR human immune-deficiency virus[tw] OR ((human immun*) AND (deficiency virus[tw])) OR acquired immunodeficiency syndrome[tw] OR acquired immunedeficiency syndrome[tw] OR acquired immuno- deficiency syndrome[tw] OR acquired immune-deficiency syndrome[tw] OR ((acquired immun*) AND (deficiency syndrome[tw])) OR "sexually transmitted diseases, viral"[MH])) OR (Antiretroviral Therapy, Highly Active[MeSH] OR Anti-Retroviral Agents[MeSH] OR Antiviral Agents[MeSH:noexp] OR ((anti) AND (hiv[tw])) OR antiretroviral*[tw] OR ((anti) AND (retroviral*[tw])) OR HAART[tw] OR ((anti) AND (acquired immunodeficiency[tw])) OR ((anti) AND (acquired immunedeficiency[tw])) OR ((anti) AND (acquired immuno-deficiency[tw])) OR ((anti) AND (acquired immune-deficiency[tw])) OR ((anti) AND (acquired immun*) AND (deficiency[tw]))) | 330134 |
| **#2** | Search randomised controlled trial[pt] AND ORcontrolled clinical trial[pt] OR randomised controlled trials[mh] OR random allocation[mh] OR double-blind method[mh] OR singleblind method[mh] OR clinical trial[pt] OR clinical trials[mh] OR ("clinical trial"[tw]) OR ((singl*[tw] OR doubl*[tw] OR trebl*[tw] OR tripl*[tw]) AND (mask*[tw] OR blind*[tw])) OR (placebos[mh] OR placebo*[tw] OR random*[tw] OR research design[mh:noexp] OR comparative study[mh] OR evaluation studies[mh] OR follow-up studies[mh] OR prospective studies[mh] OR control*[tw] OR prospectiv*[tw] OR volunteer*[tw]) NOT (animals[mh] NOT human[mh]) | 3637098 |
| **#3** | Search ((((((Comparative Study[MeSH Terms]) OR Prospective Studies[MeSH Terms]) OR Follow-Up Studies[MeSH Terms]) OR Cohort Studies[MeSH Terms]) OR Epidemiologic Studies[MeSH Terms]) OR Longitudinal Studies[MeSH Terms]) OR control* OR prospectiv* AND studies[Text Word] | 1872435 |
| **#4** | Search nevirapine OR NVP OR viramune OR numune | 6768 |
| **#5** | Search efavirenz OR sustiva OR stocrin OR EFV OR EFZ | 2833 |
| **#6** | #1 AND # 4 AND #5 AND #2 | 344 |
| **#7** | #1 AND # 4 AND #5 AND #3 | 242 |

| Cochrane Central Register **of Controlled** Trials ***(***Central***)*** | |
| --- | --- |
| **Search efavirenz AND nevirapine ( search all text)** | **73** |

| **Embase search string results** | | |
| --- | --- | --- |
| **#1** | hiv.mp. or Human immunodeficiency virus/ | 256402 |
| **#2** | acquired immune deficiency syndrome/ or antiretrovirus agent/ or Human immunodeficiency virus infection/ or Human immunodeficiency virus 1/ or hiv 1.mp. or Human immunodeficiency virus/ | 333772 |
| **#3** | hiv-1.mp. or Human immunodeficiency virus 1/ | 83927 |
| **#4** | AIDS.mp. or acquired immune deficiency syndrome/ | 185520 |
| **#5** | antiretroviral*.mp. or highly active antiretroviral therapy/ or anti human immunodeficiency virus agent/ | 59405 |
| **#6** | 1 or 2 or 3 or 4 or 5 | 396857 |
| **#7** | random*.mp. | 876837 |
| **#8** | "random*".ti,ab. | 760574 |
| **#9** | clinical trial/ | 876924 |
| **#10** | controlled clinical trial/ or random* controlled trial.mp. | 485486 |
| **#11** | randomi?sed controlled trial$.tw. | 21339 |
| **#12** | single blind procedure.mp. or single blind procedure/ | 16134 |
| **#13** | cluster random*.mp. | 3656 |
| **#14** | double blind procedure.mp. or double blind procedure/ | 114445 |
| **#15** | #7 or #8 or #9 or #10 or #11 or #12 or #13 or #14 | 1421608 |
| **#16** | comparative cohort.mp. | 282 |
| **#17** | cohort analysis/ | 126080 |
| **#18** | Comparative study.mp. or comparative study/ | 733403 |
| **#19** | (non-randomised study or non-randomized study).mp. | 1258 |
| **#20** | prospective study.mp. or prospective study/ | 263204 |
| **#21** | #16 or #17 or #18 or #19 or #20 | 1070419 |
| **#22** | efavirenz.mp. or efavirenz/ | 11541 |
| **#23** | (sustiva or stocrin).mp. [mp=title, abstract, subject headings, heading word, drug trade name, original title, device manufacturer, drug manufacturer, device trade name, keyword] | 1099 |
| **#24** | (efv or efz).mp. [mp=title, abstract, subject headings, heading word, drug trade name, original title, device manufacturer, drug manufacturer, device trade name, keyword] | 798 |
| **#25** | #22 or #23 or #24 | 11730 |
| **#26** | nevirapine.mp. or nevirapine/ | 12494 |
| **#27** | nvp.mp. | 2397 |
| **#28** | (viramune or nevimune).mp. [mp=title, abstract, subject headings, heading word, drug trade name, original title, device manufacturer, drug manufacturer, device trade name, keyword] | 1025 |
| **#29** | #26 or #27 or #28 | 14172 |
| **#30** | #6 AND #15 AND #25 AND #29 | 1673 |
| **#31** | #6 AND #21 AND #25 AND #30 | 616 |
