# Supplementary material for: Outcomes for Efavirenz versus Nevirapine-Containing Regimens for Treatment of HIV-1 Infection: A Systematic Review and Meta-Analysis
Source: PLoS One. 2013 Jul 22;8(7):e68995. doi: 10.1371/journal.pone.0068995 (PMC3718822; doi:10.1371/journal.pone.0068995)
Supplement: Table S2 — Risk of bias assessment. (DOCX) [file pone.0068995.s002.docx]

**Table S2:** **Risk of bias assessment**

| **Quality review and risk of bias^[[1]](#footnote-1)^** | | | | | | | | | | | | | | | | | | | | | | |
| --- | --- | --- | --- | --- | --- | --- | --- | --- | --- | --- | --- | --- | --- | --- | --- | --- | --- | --- | --- | --- | --- | --- |
| **Study** | | **Random sequence generation (selection bias)** | | **Allocation concealment (selection bias)** | | **Blinding of participants and personnel (performance bias)** | **Blinding of outcome assessment (detection bias)** | **Selective reporting (reporting bias)** | **Comparability of baseline groups (similar)** | **Details of groups difference compared to EFV** | **ITT principle violated** | **LTFU (attrition) stated and <20% (note %)** | | | | **LTFU: NVP** | | **LTFU: EFV** | | **Other biases/comments** | | |
| **RCTS** | |  | |  | |  |  |  |  |  |  |  | | |  | |  | | |  | |  |
| **Gaytan *et al* (2004)** | | Y | | N | | N | N | Y | Y | no difference | Y | N | | | NR | | NR | | | NR | |  |
| **Nunez *et al* (2002) SENC** | | Y | | N | | N | N | N | Y | no difference | N | Y 8/67 (12%) | | | 5/36 | | 3/31 | | | Prednisolone given in lead in dose, 400mg OD of NVP | |  |
| **Landman *et al* (2011) DAYANA** | | NR | | N | | N | N | N | NR | ABSTRACT ONLY | NR | Y 1/ 119 (0.8%) | | | NR | | NR | | | NR | |  |
| **Wit *et al* (2007) 2NN** | | Y | | Y | | N | N | Y | Y | no difference ^[[2]](#footnote-2)^ | N | NR | | | NR | | NR | | | Authors received grants from pharmaceutical companies, study funded by Boehringer-Ingelhiem | |  |
| **Swaminathan *et al* (2011)** | | Y | | y | | NR | N | N | Y | no difference | N | Y 9/122 (7,4%) | | | 3/57 | | 3/59 | | | DSMB halted trial at 2^nd^ interim analysis, 400mg OD of NVP | |  |
| **Manosuthi *et al* (2009) N2R** | | NR | | NR | | N | N | N | Y | no difference | N | Y 9/42 (6%) | | | 4/71 | | 5/71 | | | NR | |  |
| **Wester *et al* (2010) TSHEPO** | | Y | | NR | | N | N | Y | Y | no difference | N | Y 54/650 (8.3%) | | | NR | | NR | | | Funding from Bristol Meyers Squibb, change in definitions after majority reached study follow-up, study underpowered | |  |
| **Bonnet *et al* (2013) CARINEMO** | | NR | | NR | | N | N | N | NR | no difference | N | NR | | | NR | | NR | | | NR | |  |
| **van den Berg-Wolf *et al* (2008) CPCRA/INSIGHT** | | Y | | NR | | N | N | Y | Y | no difference | N | NR | | | NR | | NR | | | Authors received grants from pharmaceutical companies | |  |
| **Matteelli et al (2013)** | | NR | | NR | | NR | NR | Y | Y | no difference | NR | Y (1/69) | | | NR | | NR | | | NR | |  |
| **Study** | **Random sequence generation (selection bias)** | | **Allocation concealment (selection bias)** | | **Blinding of participants and personnel (performance bias)** | | **Blinding of outcome assessment (detection bias)** | **Selective reporting (reporting bias),** | **Comparability of baseline groups (similar)** | **Details of groups difference compared to EFV** | | | **ITT principle violated** | **LTFU (attrition) stated and <20% (note %)** | | | | **NVP** | **EFV** | |  |  |
| **PROSPECTIVE COHORTS** |  | |  | |  | |  |  |  |  | | |  |  | | | |  |  | |  |  |
| **Keiser *et al* (2002)** | NR | | NR | | NR | | NR | N | N | difference in race, CD4 count | | | N | NR | | | | NR | NR | |  |  |
| **Annan *et al* (2009)** | NR | | NR | | NR | | NR | N | N | NVP lower baseline VL, higher CD4, more females, difference in NRTI backbone | | | N | NR | | | | NR | NR | |  |  |
| **Reekie *et al* (2011) EUROSIDA** | NR | | NR | | NR | | NR | N | N | NVP higher CD4 and lower VL, small diff in NRTI backbone | | | Y | NR | | | | NR | NR | |  |  |
| **Hamers *et al* (2012)** | NR | | NR | | NR | | NR | N | N | NR | | | N | NR | | | | NR | NR | |  |  |
| **Fox *et al* (2012) IDEA** | NR | | NR | | NR | | NR | N | N | NR | | | N |  | | | | NR | NR | |  |  |
| **Cortes *et al* (2010)** | NR | | NR | | NR | | NR | N | N | NVP had less advanced disease, higher median CD4, but similar VL | | | NR | NR | | | | NR | NR | |  |  |
| **Castelnuovo *et al* (2011)** | NR | | NR | | NR | | NR | N | N | NR | | | NR | Y 13/474 (2,7%) | | | | NR | NR | |  |  |
| **ART-CC (2006)** | NR | | NR | | NR | | NR | N | N | NR | | | N | NR | | | | NR | NR | |  |  |
| **Mugavero *et al* (2008) ART CC** | NR | | NR | | NR | | NR | N | N | NVP less commonly prescribed, similar for the rest | | | N | NR | | | | NR | NR | |  |  |
| **HIV CAUSAL (2012)** | NR | | NR | | NR | | NR | N | N | NVP more females, higher CD4, lower viral load | | | N | Y 0.5% | | | | NR | NR | |  |  |
| **Perez-Elias *et al* (2005)** | NR | | NR | | NR | | NR | N | N | NR | | | N | NR | | | | NR | NR | |  |  |
| **Hartmann *et al* (2005) NEEF** | NR | | NR | | NR | | NR | N | N | no difference | | | NR | Y 113/662 (17%)^[[3]](#footnote-3)^ | | | | NR | NR | |  |  |
| **Manfredi *et al* (2004)** | NR | | NR | | NR | | NR | N | N | no difference | | | N | NR | | | | NR | NR | |  |  |
| **de Beaudrap *et al* ( 2008) ANRS 1215/1290** | NR | | NR | | NR | | NR | N | N | NVP more females, lower VL and more on CTX^[[4]](#footnote-4)^ | | | NR | NR | | | | NR | NR | |  |  |
| **Wallis *et al* (2012) CIPRA-SA** | Y | | N | | N | | N | N | N | NR | | | N | NR | | | | NR | NR | |  |  |

| **Study** | **Random sequence generation (selection bias)** | **Allocation concealment (selection bias)** | **Blinding of participants and personnel (performance bias)** | **Blinding of outcome assessment (detection bias)** | **Selective reporting (reporting bias),** | **Comparability of baseline groups (similar)** | **Details of groups difference compared to EFV** | **ITT principle violated** | **LTFU (attrition) stated and <20% (note %)** | **NVP** | **EFV** |
| --- | --- | --- | --- | --- | --- | --- | --- | --- | --- | --- | --- |
| **RETROSPECTIVE COHORTS** |  |  |  |  |  |  |  |  |  |  |  |
| **Darin *et al* (2010) PEPFAR Nigeria** | NR | NR | NR | NR | Y | N | no difference | NR | Y 1663/8418 (19.8%) | 1162/6254 | 501/2164 |
| **von Giesen *et al* (2003)** | NR | NR | NR | NR | N | N | NVP had less TB infected patients, more females | N | NR | NR | NR |
| **Shipton *et al* (2009) Masa** | y | NR | NR | NR | N | N | NR | NR | NR | NR | NR |
| **Cescon *et al* (2010) CANOC** | NR | NR | NR | NR | N | N | NR | NR | NR | NR | NR |
| **Villar *et al* (2011)** | NR | NR | NR | NR | N | N | approx 98% MALE in both arms | not clear | Y 5/63 (8%) | 0/13 | 5/50 |
| **Braithwaite *et al* (2007) 'Veterans'** | NR | NR | NR | NR | N | N | NVP less advanced stage, higher CD4, lower VL and less with AIDS | N | Nr | NR | NR |
| **Cozzi-Lepri *et al* (2002) ICONA** | NR | NR | NR | NR | N | N | NVP more females, higher CD4 | NR | Nr | NR | NR |
| **Manosuthi *et al* (2004)** | NR | NR | NR | NR | N | N | no difference | Y | Y 11/66 (17%) | 5/30 | 6/36 |
| **Manosuthi *et al* (2008)** | NR | NR | NR | NR | N | N | NVP younger, more females, less immunosuppressed, lower viral load, started treatment earlier calendar time | N | Y 16/188 (8,5%) | 6/111 | 10/77 |
| **Nachega *et al* (2008)** | NR | NR | NR | NR | N | N | TDF/XTC/EFV more males, lower CD4 and TB. AZT/3TC/NVP and TDF/ XTC/NVP more females. Higher adherence on TDF/XTC/EFV  regardless of NNRTI used. | N | Y 454/2817 (16%) | 203/995 | 251/1822 |
| **Amoroso *et al* (2012) AIDS Relief** | Y | NR | NR | NR | N | N | NR | NR | NR | NR | NR |
| **Bock (2010)** | NR | NR | NR | NR | NR | N | NR | NR | NR | NR | NR |
| **Chung and Fichtenbaum (2002)** | NR | NR | NR | NR | N | NR | Abstract only | NR | NR | NR | NR |
| Abbreviations: EFV efavirenz NVP nevirapine TDF tenofovir NRTI nucleoside reverse transcriptase inhibitor NNRTI non-nucleoside reverse transcriptase inhibitor ITT Intention to treat LTFU Lost to follow up Y Yes N NO NR not reported | | | | | | | | | | | |

1. All cohorts were followed up for a sufficient time of at least 6 months [↑](#footnote-ref-1)
2. As reported in van Leth^5^ 2NN study report [↑](#footnote-ref-2)
3. For entire cohort including non-naïve patients N [↑](#footnote-ref-3)
4. cotrimoxazole chemoprophylaxis [↑](#footnote-ref-4)
