# Supplementary material for: Outcomes for Efavirenz versus Nevirapine-Containing Regimens for Treatment of HIV-1 Infection: A Systematic Review and Meta-Analysis
Source: PLoS One. 2013 Jul 22;8(7):e68995. doi: 10.1371/journal.pone.0068995 (PMC3718822; doi:10.1371/journal.pone.0068995)
Supplement: Table S3 — Sensitivity analysis results. (DOCX) [file pone.0068995.s003.docx]

**Table S3: Sensitivity analysis results**

| **Summary of sensitivity analysis differentiated by NVP dosages** | | |
| --- | --- | --- |
| **Outcome** | Strict comparison of studies: EFV vs. NVP 200mg twice daily | Comparison of studies of EFV 600mg once daily to all studies of NVP 200mg twice daily and studies of NVP 400mg once daily ^^[[1]](#footnote-1)^^ |
| **Virologic success** | RR 1.04 [1.00-1.09] p = 0.05, p for heterogeneity=0.40 I²=4%. | RR 1.06 [1.00, 1.12] p = 0.06, p for heterogeneity = 0.21 I² = 26% |
| **Virologic failure** | RR 0.83 [0.73 - 0.94] p = 0.004,p for heterogeneity = 0.98 I² = 0% | RR 0.82 [0.73, 0.93] p = 0.0002, p for heterogeneity = 0.96 I² = 0% |
| **Mortality** | RR 0.94 [0.59, 1.49] p = 0.79, p for heterogeneity = 0.32 I²=41% | RR 0.79 [0.42, 1.49] p = 0.47, p for heterogeneity = 0.17 I² = 12% |
| **Treatment termination** | RR0.71 [0.43, 1.17] p = 0.18, p for heterogeneity <0.002 I² = 80% | RR 0.76 [0.48, 1.20] p = 0.24, p for heterogeneity = 0.004 I² = 74% |

| **Summary of sensitivity analysis differentiated by viral load cut off** | | | |
| --- | --- | --- | --- |
| **Virologic failure RCTs** | >50 copies/ml: RR 0.85 [0.73, 0.99] p=0.04 p for heterogeneity =0.93 I² = 0% | >400 copies/ml: RR 0.85 [0.73, 0.99] p=0.04p for heterogeneity =0.85I² = 0% | >1000 copies/ml: RR 0.86 [0.73, 1.00] p=0.06 p for heterogeneity =0.78 I² = 0% |
| **Virologic failure observational studies** | >50 copies/ml: RR 0.65 [0.59, 0.71]p=0.00002p for heterogeneity =0.02 I² = 54% | >400 copies/ml: RR 0.65 [0.59, 0.71]p=0.00001 p for heterogeneity =0.01 I² = 57% | >1000 copies/ml: RR 0.73 [0.66, 0.80] p=0.00001 p for heterogeneity =0.4 I² = 0% |
| **Virologic success RCTs** | <50 copies/ml: RR 1.04 [1.00, 1.08] p=0.05p for heterogeneity =0.67 I² = 0% | <400 copies/ml: RR 1.04 [1.00, 1.08] p=0.06p for heterogeneity =0.73 I² = 0% | - |
| **Virologic success observational studies** | <50 copies/ml RR 1.06 [1.02, 1.11]p=0.005p for heterogeneity =0.28 I² = 20% | <400 copies/ml RR 1.06 [1.00, 1.12] p=0.04p for heterogeneity =0.0002 I² = 68% | - |

1. [↑](#footnote-ref-1)
